# Fabrication of 3D-Printed Fish-Gelatin-Based Polymer Hydrogel Patches for Local Delivery of PEGylated Liposomal Doxorubicin

**DOI:** 10.3390/md18060325

**Published:** 2020-06-20

**Authors:** Jin Liu, Tatsuaki Tagami, Tetsuya Ozeki

**Affiliations:** Drug Delivery and Nano Pharmaceutics, Graduate School of Pharmaceutical Sciences, Nagoya City University, 3-1 Tanabe-dori, Mizuho-ku, Nagoya, Aichi 467-8603, Japan; ciwujialiujin@gmail.com (J.L.); tagami@phar.nagoya-cu.ac.jp (T.T.)

**Keywords:** 3D bioprinter, fish gelatin methacryloyl (F-GelMA), liposome, nanomedicine, controlled drug release

## Abstract

3D printing technology has been applied to various fields and its medical applications are expanding. Here, we fabricated implantable 3D bio-printed hydrogel patches containing a nanomedicine as a future tailored cancer treatment. The patches were prepared using a semi-solid extrusion-type 3D bioprinter, a hydrogel-based printer ink, and UV-LED exposure. We focused on the composition of the printer ink and semi-synthesized fish gelatin methacryloyl (F-GelMA), derived from cold fish gelatin, as the main component. The low viscosity of F-GelMA due to its low melting point was remarkably improved by the addition of carboxymethyl cellulose sodium (CMC), a pharmaceutical excipient. PEGylated liposomal doxorubicin (DOX), as a model nanomedicine, was incorporated into the hydrogel and liposome stability after photo-polymerization was evaluated. The addition of CMC inhibited particle size increase. Three types of 3D-designed patches (cylinder, torus, gridlines) were produced using a 3D bioprinter. Drug release was dependent on the shape of the 3D-printed patches and UV-LED exposure time. The current study provides useful information for the preparation of 3D printed nanomedicine-based objects.

## 1. Introduction

Liposomes are biocompatible lipid vesicles extensively used as drug carriers for nanomedicine [1,2,3]. Liposomes can encapsulate anticancer drugs with a narrow therapeutic window and reduce non-specific drug distribution in organ tissues. For example, the anticancer drug doxorubicin (DOX) is a broad-spectrum anticarcinogen used to treat breast cancer, bladder cancer, Kaposi’s sarcoma, lymphoma and acute lymphocytic leukemia, but exhibits serious and irreversible cardiotoxicity. PEGylated liposomal DOX, Doxil^®^, was the first nanomedicine approved by the US Food and Drug Administration for cancer treatment and has remarkably reduced cardiotoxicity compared to free DOX solution [4]. PEGylating liposomes can prolong their circulation time in blood [5], resulting in their passive accumulation in cancer tissue, called the enhanced permeability and retention effect [6].

The implantation of drug-loaded devices, and the application of nanomedicine, are useful strategies for controlling and maintaining the required drug concentration in the target tissue. Compared to systemic drug delivery, local drug delivery allows the local, surgical administration of drugs directly to cancer tissue. Drug-loaded polymer nanoparticles and radio-sensitizing nanoparticles are attractive options for the treatment of local tumors (e.g., brain tumor) [7]. Nanomaterial implantation has been investigated for preventing infection after orthopedic surgery, and for diagnosis and cancer therapy [8]. There are several methodologies for the implantation of drug-loaded devices and nanomedicines for drug delivery. For example, cast implants, injectable implants, and 3D printed implants for implantation into bone and cancer tissues [9] utilize the properties of biopolymers, biomaterials and nanocarriers.

3D printing technologies are widely used in manufacturing and their medical applications are expanding. Computer-aided design is used to translate two-dimensional images such as X-ray, nuclear magnetic resonance and computer tomography images into 3D data and to fabricate medical devices such as surgical guides, artificial arms and legs, wound dressings and organ models [10,11,12]. 3D printing methodologies include stereolithography, inkjet printing, fused deposition modelling, semi-solid extrusion printing and selective laser sintering, and have been applied to different materials such as powders, pastes, gels and resins. 3D printed implants and medicines have been approved by the US Food and Drug Administration [13], and 3D printed tablets are being investigated [14]. Several clinical trials involving 3D printing are in progress [15].

3D bioprinting has been used to generate designed objects for tissue engineering and pharmaceutical applications using highly printable and stable bioinks [16,17]. Semi-solid extrusion-type 3D printers use pastes and hydrogels as bioinks extruded from the nozzle by air pressure. The extruded materials are deposited layer-by-layer to create the intended structure. 3D bioinks contain cells, growth factors, biomaterials or nanoparticles, depending on the application. Implants should mimic the characteristics of natural tissues. Many types of polymers are used, including natural polymers (collagen, gelatin and alginate), semi-synthetic polymers (methacrylated hyaluronic acid, gelatin methacryloyl (GelMA), PEG diacrylate and norbornene-functionalized hyaluronic acid [18]), and synthetic polymers (hydroxypropyl methylcellulose, polyacrylic acid, poly(lactic-co-glycolic) acid and polycaprolactone [19]).

**Scheme 1:**
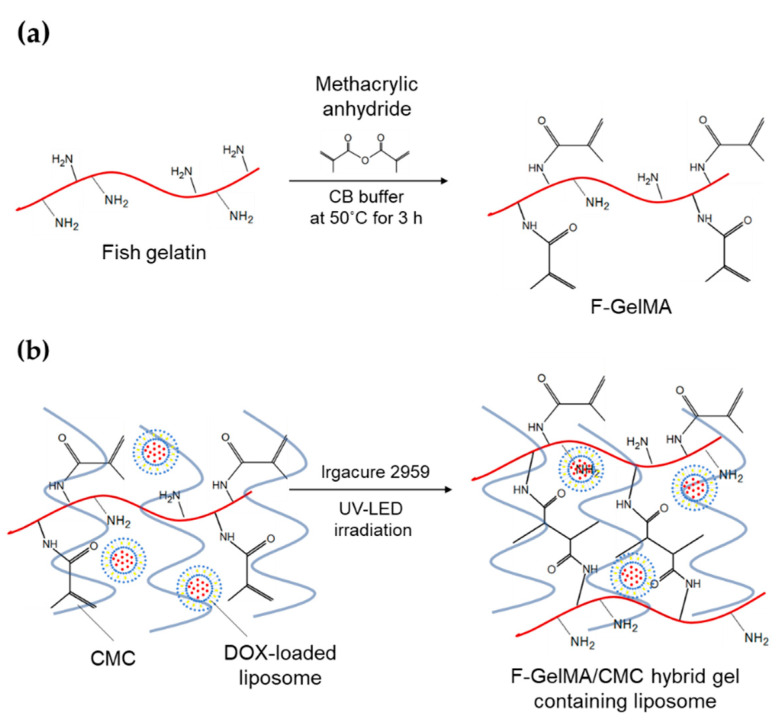
(**a**) Synthesis of fish gelatin methacryloyl (F-GelMA). (**b**) Hybrid gel of cross-linked F-GelMA and carboxymethyl cellulose sodium (CMC) containing PEGylated liposome. The reaction scheme was prepared in previous studies [20,21].

In the current study, we generated liposomal patches for implantation in cancer tissue (Scheme 1) using a 3D bioprinter. We used a hydrogel containing semi-synthetic fish-gelatin polymer (fish gelatin methacryloyl, F-GelMA) to entrap DOX-loaded PEGylated liposomes. Fish gelatin is inexpensive and faces few personal or religious restrictions [22]. F-GelMA is generated by reacting cold water fish gelatin with methacrylic anhydride and forms photopolymerized hydrogels in the presence of a photoinitiator and UV irradiation. F-GelMA has been investigated by several groups for use in tissue engineering and biofabrication [23,24,25,26] but it remains unclear whether its characteristics are useful as a drug delivery system [27]. F-GelMA is rarely bioprinted because of its low viscosity and rapid polymerization. In the present study, we increased the viscosity of F-GelMA by incorporating a pharmaceutical additive into the bioink. The bioink and 3D printed products containing liposomes were characterized.

## 2. Results and Discussion

**Figure 1:**
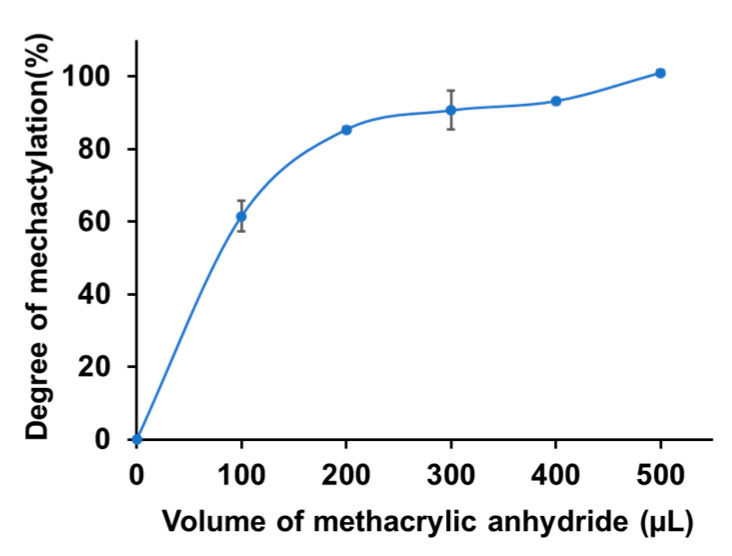
Rate of synthesis of F-GelMA. The data represent the mean ± SD (n = 3).

In the present study, we used F-GelMA, a cold water fish gelatin-based biocompatible polymer. Gelatins are divided into two categories: cold-adapted gelatin and warm-adapted gelatin. Zaupa et al. described the technical advantages of cold-adapted gelatins for biofabrication applications over warm-adapted gelatins by comparing their structural and molecular features [24]. Shirahama et al. described a facile one-pot GelMA synthesis method [28]; however, it is not compatible with F-GelMA derived from cold water fish gelatin because the gelatin sediments during dialysis. Following previous study [29], here we succeeded in synthesizing F-gelatin, as shown in Figure 1. F-gelatin reacts with methacrylic anhydride, with one methacrylic anhydride molecule reacting per free amino group. The solution pH affects the protonation of free amino groups on gelatin, and thus we used sodium carbonate-bicarbonate buffer to provide a pH of around 9.0. The method based on 2,4,6-trinitrobenzenesulfonic acid sodium salt dihydrate (TNBS) was used to quantify the degree of methacrylation. A methacrylic anhydride to F-gelatin ratio of 0.5 mL to 1 g (*v*/*w*) provided a yield of 101.0% ± 0.8% (Figure 1), indicating the extent of substitution of free amino groups in gelatin and the F-GELMA chains and a higher degree of GelMA methacrylation than that reported by Yoon et al. (91.4 ± 3.1%) [23].

The melting point of fish gelatin is very different from that of porcine and bovine gelatin. Wang et al. demonstrated that the thermal stability (i.e., low variation in viscosity with temperature) of fish skin greatly benefits many biofabrication applications using microfluidic droplet generation compared with porcine gelatin [26]. However, the viscosity of F-GelMA hydrogel derived from fish gelatin is very low at room temperature and we could not measure its viscosity. The low melting point of fish gelatin is due to its composition of amino acids, especially a low proportion of hydroxyproline and proline [30]. This low viscosity makes the gelatin unsuitable for 3D bioprinting because the sample leaks from the nozzle and the 3D printed object collapses under its own weight. We thus used a pharmaceutical excipient to raise the viscosity of F-GelMA-based formulations. For example, alginate is biodegradable and has high viscosity and is often used as pharmaceutical excipient with GelMA [25]. Biodegradability and high viscosity are necessary characteristics for a pharmaceutical excipient used in bio-3D printing.

**Figure 2:**
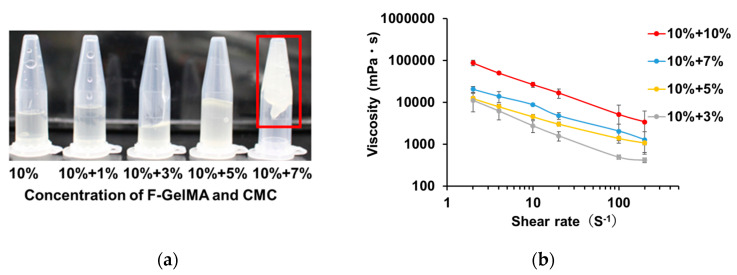
Viscous properties of drug formulations used as printer inks. (**a**) The appearance of F-GelMA hydrogels containing different concentrations of CMC. (**b**) The viscosity profiles of F-GelMA hydrogels containing different concentrations of CMC. The data represent the mean ± SD (n = 3).

Carboxymethyl cellulose sodium (CMC) is a cellulose derivative often used as an excipient in the pharmaceutical and food industries to thicken jellies and syrup, to form suspensions, as a binder in tablet preparations, and as an emulsifier for ointments, creams and lotions due to its water absorbency and high viscosity [31]. In the current study, we prepared hybrid hydrogel formulations comprising F-GelMA and CMC and investigated their viscous properties. As shown in Figure 2a,b, the addition of CMC increased the viscosity of hydrogel in a CMC concentration-dependent manner. Log scale differences in viscosity were confirmed visually. A polymer-dependent increase in viscosity was previously observed using hydroxypropyl methylcellulose to prepare 3D printer oral films [32]. To our knowledge, this is the first study using CMC to prepare bioink-containing liposomes.

**Figure 3:**
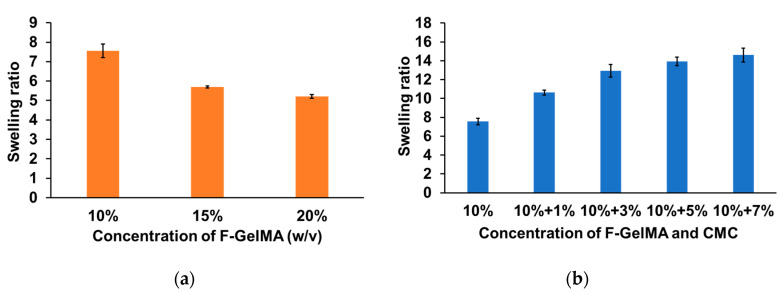
Swelling properties of hydrogels after photopolymerization. (**a**) Swelling ratio of different concentrations of F-GelMA. (**b**) Swelling ratio of mixed hydrogel (10% F-GelMA with different concentrations of CMC). The data represent the mean ± SD (n = 3).

The swelling characteristics of hydrogels are important because they affect solute diffusion and mechanical properties [33]. We fabricated F-GelMA hydrogels under different conditions (different concentrations of 10%, 15%, 20% (*w/v*) and mixed hydrogels F-GelMA (10%) + CMC (1%, 3%, 5%, 7%) (*w/v*). The swelling behavior of hydrogels depends on their structural properties, such as interactions with the solvent, cross-linking density, and hydrophilicity [34]. The swelling ratio decreased with increasing concentration of F-GelMA (Figure 3a) and increased with increasing concentration of CMC. The combination of 10% F-GelMA and 7% CMC showed the highest swelling ratio (Figure 3b).

We assessed the stabilities of the liposome formulations in 3D-printed patches by measuring the particle diameters and polydispersity index (PDI). PEGylated liposomal DOX was prepared and the average size of the liposomes was 120.5 ± 1.9 nm (Figure S1a) and the encapsulation efficiency was 99.2 ± 1.0%. PEGylated liposomal DOX was embedded within 10%, 15% or 20% F-GelMA or a mixture of F-GelMA (10%, 15%, 20%) and CMC (7%). The hydrogel formulation containing 20% F-GelMA alone had a larger particle size than that containing 20% F-GelMA + 7% CMC (Figure S1b) and the PDI of hydrogel formulations containing F-GelMA tended to be larger than those containing F-GelMA + CMC. These results suggested that PEGylated liposomal DOX in a mixture of CMC and F-GelMA is more stable than that of F-GelMA alone, stabilizing the liposomes in the hydrogel. The liposomes were dispersed in the hydrogel network formed between GelMA molecules after UV crosslinking. The phosphoric acid groups of the liposomes also formed micro-crosslinks with GelMA molecules, as described by Cheng et al., who prepared liposome-modified hydrogels stabilized by hydrogen bonding [35]. Moreover, GelMA in GelMA-CMC acts as a stable structural element, while CMC may act as a dynamic modifiable element, as suggested by Chen et al., with regards to GelMA-alginate composite hydrogel [36]. We considered that the addition of CMC could further extend crosslinking between liposomes and hydrogels.

**Figure 4:**
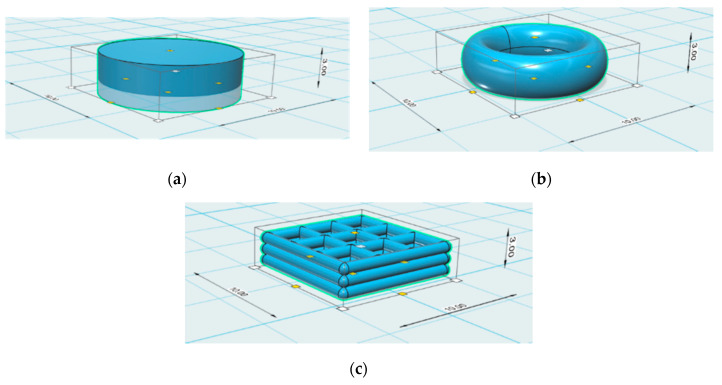
Design of the different 3D geometries: (**a**) cylinder, (**b**) torus, and (**c**) gridlines.

**Figure 5:**
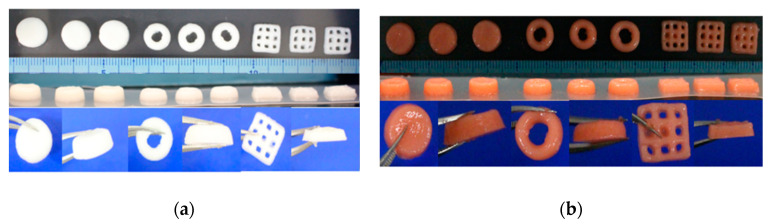
3D bioprinting of patches. Appearance of 3D-printed patches (**a**) without or (**b**) with PEGylated liposomal doxorubicin before UV irradiation. The printer ink contained 10% F-GelMA and 7% CMC.

Next, we 3D printed patches using a semi-solid extrusion-type 3D bioprinter. The printing conditions are shown in Table 1, and the 3D designs of three shapes (cylinder, torus, gridlines) are shown in Figure 4. As shown in Figure 5, the desired patch shapes were prepared using the 3D bioprinter.

We simulated in vivo environments to test the release of liposomes at 37 °C using 4-(2-hydroxyethyl)-1-piperazineethanesulfonic acid (i.e., HEPES) buffer (pH 7.45) as the dissolution medium. Several factors can affect the release rate of liposomes from patches, such as surface area, density of crosslinks (length of UV irradiation), temperature, and shaker speed. We expected that larger surface areas would induce faster drug release keeping other parameters (temperature, dissolution medium and shaker speed) fixed. Martinez et al. showed that surface area affected drug release from oral tablets prepared using a stereolithography-type 3D printer [37]. We also previously showed that surface area affects initial drug release from oral tablets prepared using a fused deposition modeling-type 3D printer [38]. In addition, we speculated that the extent of hydrogel crosslinking can affect the release of liposomes. The effect of UV exposure time on drug release behavior is not well investigated.

**Figure 6:**
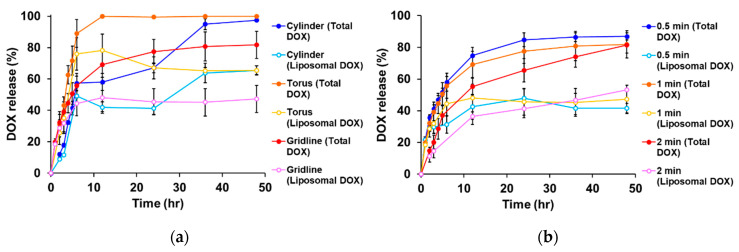
Drug release profiles of liposomal doxorubicin (DOX). (**a**) Influence of shape on drug release. The UV exposure time was set to 1 min. (**b**) Influence of UV exposure time on drug release. The gridline object was used for this experiment. The data represent the mean ± SD (n = 3).

Our investigation of drug release from different shapes of patches showed that the order of drug release was torus > gridline = cylinder (Figure 6a). Torus-type patches have a larger surface area and could dissolve quickly, whereas gridline-type and cylinder-type patches dissolved more slowly. The total DOX release rate from cylindrical patches may be irregular due to incomplete UV polymerization causing complicated release kinetics. We also evaluated the influence of patch type on the release of liposomal DOX and observed a tendency similar to that described above. In our system, gridline-type patches may be best for sustained release.

We investigated the effect of UV exposure time on drug release, as shown in Figure 6b. The order of drug release was 0.5 min ≧ 1 min > 2 min. Increasing the UV irradiation time induced increased crosslinking density in the hydrogel. The longest UV irradiation time tested (2 min) delayed DOX release (Figure 6b). The release of liposomal DOX has similar tendencies to that of total DOX. Furthermore, DOX leaked from liposomes during their release from patches, which is known by comparing the results of total DOX and liposomal DOX (Figure 6). The results shown in Figure S1b indicate that the PDI of liposomes in hydrogels increased compared to liposomes alone. Liposomes in hydrogels are unstable during release due to possible interaction with F-GelMA, as mentioned above. Understanding the network between liposomes and hydrogels is necessary for developing hydrogels to enhance the stability of liposomes. We showed that the 3D patches may exhibit controlled release when used as an implant, whereas longer sustained release is necessary to control cancer growth. Biphasic drug release was observed (Figure 6a). This is due to the initial burst release and the retention in hydrogels and liposomes in the long term. We did not find sufficient results that fit the typical model of release kinetics. We think that slower drug release from gels and liposomes can contribute to the sustained inhibitory effect on cancer growth, although future study is necessary.

## 3. Materials and Methods

### 3.1. Materials

Gelatin from cold water fish skin and methacrylic anhydride were purchased from Sigma-Aldrich (St. Louis, MO, USA). Dipalmitoyl phosphatidylcholine (DPPC), DOX hydrochloride, and 2-hydroxy-4′-(2-hydroxyethoxy)-2-methylpropiophenone (Irgacure 2959) were purchased from Tokyo Chemical Industry (Tokyo, Japan). Sepharose CL-4B was purchased from GE Healthcare Bio-Sciences (Piscataway, NJ, USA). HEPES was purchased from Nacalai Tesque (Kyoto, Japan). Cholesterol (CHOL), sodium dodecyl sulfate and TNBS were purchased from Fujifilm Wako Pure Chemical Corporation (Osaka, Japan). *N*-(Carbonyl-methoxypolyethyleneglycol 2000)-1,2-distearoyl-sn-glycero-3-phosphoethanolamine (mPEG2000-DSPE) was kindly provided by Nippon Fine Chemical Co. Ltd. (Osaka, Japan). CMC was provided by Gotoku Chemical Company, Ltd. (Tokyo, Japan).

### 3.2. Synthesis of F-GelMA

F-GelMA was synthesized as previously described with minor modifications [39]. In brief, 10 g of fish gelatin was dissolved in 100 mL sodium carbonate-bicarbonate buffer (0.1 M, pH 9.0), and different volumes (1.2 mL, 1 mL, 0.8 mL, 0.4 mL, 0.2 mL) of methacrylic anhydride were added 6 times every 30 times and the pH was adjusted to 9. Methacrylic anhydride was added at a rate of 0.5 mL/min to the fish gelatin solution with stirring at 50 °C and reacted for 3 h. The mixture was diluted 5-fold with distilled water to stop the reaction, and dialyzed using dialysis tubing (MWCO 12,000–14,000, Thermo Fisher Scientific, Waltham, MA, USA) for 48 h at 50 °C to remove unreacted methacrylic anhydride and methacrylic acid byproducts. The solution was lyophilized (FD-1000; EYELA, Tokyo, Japan) to generate a white porous foam and stored at 4 °C until use. Lyophilized F-GelMA was dissolved in HEPES buffer containing 0.5% (*w/v*) Irgacure 2959 as a photosensitizer at 80 °C, and used to fabricate hydrogels.

### 3.3. Evaluation of the Degree of Methacrylation of F-GelMA

The degree of methacrylation of F-GelMA was measured using a TNBS method developed by Habeeb, as previously described [29,40]. Briefly, the freeze-dried F-GelMA prepared above was dissolved in 1% sodium dodecyl sulfate solution at 50 °C to provide a 2 mg/mL solution, then 1 mL of 4% NaHCO_3_ (pH = 8.5) and 1 mL of 0.1% TNBS solution was added to 1 mL of sample solution and reacted at 50 °C for 60 min. The reaction was stopped and stabilized by adding 2 mL of 0.1 mol/L HCl to each sample and the absorbance at 335 nm was measured using a microplate reader (Nivo 3S; PerkinElmer Inc., Waltham, MA, USA). The degree of methacrylation was calculated using Equation (1):

Degree of methacrylation = 1 − (free amino groups in F-GelMA)/(free amino groups in fish gelatin) × 100%(1)

### 3.4. Hydrogel Swelling Test after Photopolymerization

Drug formulation (200 μL) comprising F-GelMA (10%, 15%, 20% (*w/v*)) solution or a hydrogel mixture of F-GelMA (10%) and CMC (1%, 3%, 5%, 7% (*w/v*)) was transferred into the wells of a 96-well plate and exposed to UV-LED light (365 nm) from a 3D bioprinter (INKREDIBLE; CELLINK, Gothenburg, Sweden) for 60 s for polymerization, then each sample was placed in HEPES buffer at 37 °C for 24 h. Excess HEPES buffer was removed and the weight of the swollen hydrogel was recorded. Samples were lyophilized and the dried polymer was weighed. The mass swelling ratio was calculated using Equation (2):

Swelling ratio = mass of swollen hydrogel/mass of dried polymer(2)

### 3.5. Viscosity Test of Drug Formulation for Use as a Printer Ink

Drug formulation (0.5 mL) comprising F-GelMA solution (10%, (*w*/*v*)) or a hydrogel mixture of F-GelMA (10%) and CMC (3%, 5%, 7%, 10% (*w*/*v*)) was placed in a viscometer cup and the cup was set in a cone-plate type DV2T viscometer (Brookfield Viscometers, Brookfield, Middleboro, MA, USA). The rotation speed was increased from 0.1 to 200 rpm every 30 s, and the viscosity of the hydrogel at each speed was measured.

### 3.6. Preparation of DOX-Loaded PEGylated Liposomes

Liposomes were prepared by a remote loading method to provide a different pH in the interior and exterior phases by changing the ion charge of the therapeutic drug, thus increasing encapsulation efficiency [41]. The liposomes consisted of DPPC, CHOL and DSPE-PEG2000 in the molar ratio DPPC:CHOL:DSPE-PEG2000 = 55:45:10. All of the lipid components and DSPE-PEG2000 were dissolved in isopropanol in a test tube (total volume 1 mL). The solvent was evaporated by purging with nitrogen at 65 °C and the residue was further dried under reduced pressure. A thin lipid film formed on the inside of the test tube was hydrated with 1 mL ammonium sulfate solution (pH 4) and vortexed while heating in a water bath. The suspension was extruded by filtering ten times through a polycarbonate membrane (200 nm pores), then ten times through two stacked membranes (100 nm pores) to control the size of the liposomes. The liposome dispersion was injected into a dialysis cassette (10,000 MWCO; Thermo Scientific, Rockford, IL, USA) and dialyzed for 24 h against HEPES buffer (pH 7.45) with one buffer change after 12 h. DOX aqueous solution (20 mg/mL) was added to the mixture (drug/lipid = 1/5, *w/w*) and incubated in a water bath at 65 °C for 1 h. The mean diameter of the DOX-loaded PEGylated liposomes was measured using a dynamic light scattering apparatus (ZetaSizer Nano series; Malvern Instruments, Worcestershire, UK).

### 3.7. Encapsulation Efficiency of DOX into the Liposomes

The encapsulation efficiency of DOX into the liposomes was determined as previously described with modification [41,42]. As a typical experiment, a sample containing DOX-loaded liposomes and unencapsulated DOX was separated using gel chromatography by loading on a Sepharose CL-4B gel column equilibrated with HEPES buffer, and the liposome fraction was collected. The liposomes were dissolved by adding organic solvent (methanol:isopropanol = 1:3) at 4:1 (*v/v*) organic solvent:liposomes and then diluted with HEPES buffer. The DOX concentration was determined from its fluorescence intensity using a microplate reader (ARVO series, PerkinElmer Inc., Waltham, MA, USA; ex/em, 480/595 nm) and the encapsulation efficiency was calculated using Equation (3):

Encapsulation efficiency% = (amount of drug loaded in liposomes)/(amount of drug used) × 100%(3)

### 3.8. Stability Test of Liposomes Following Photopolymerization of the Hydrogel

F-GelMA solution (10%, 15%, 20% (*w/v*)) or a hydrogel mixture of F-GelMA (10%, 15%, 20% (*w/v*)) and CMC (7% (*w/v*)) containing 0.5% (*w/v*) Irgacure 2959 was mixed with DOX-loaded liposomes at a ratio of F-GelMA:DOX of 1 g: 1 mg (*w/w*). The samples were transferred to the wells of a 96-well plate and exposed to UV-LED light from a 3D bioprinter (CELLINK) for 60 s, then diluted with 10 mL HEPES buffer at 37 °C for 30 min. The mean diameter of the PEGylated liposomal DOX particles was measured using a dynamic light scattering apparatus (Malvern Instruments, Malvern, UK).

### 3.9. 3D Bioprinting

A hydrogel mixture of F-GelMA (10% (*w/v*)) and CMC (7% (*w/v*)) containing 0.5% (*w/v*) Irgacure 2959 was mixed with DOX-loaded liposomes at a ratio of F-GelMA:DOX of 1 g:1 mg to prepare printer ink. Three 3D geometries (cylinder, torus, gridlines) were designed using Autodesk 123D software, and the g-code to program the printing conditions was generated using Slic3r software. The printer ink was loaded into the CELLINK bioprinter syringe and the object was 3D printed using the conditions shown in Table 1. The 3D-printed patches were solidified by photopolymerization by exposure to the CELLINK UV-LED light for 1 min, 1.5 min or 2 min.

### 3.10. In Vitro Release of DOX-Loaded Liposomes from 3D-Printed Patches

As a typical experiment, 3D-printed patches (cylinder, torus, gridline) prepared in Section 3.9 were exposed to UV for set times. The patches were placed in 10 mL HEPES buffer (pH 7.4) and shaken using a thermo bath shaker (SN-60SD; Nissinrika, Tokyo, Japan) at 37 °C. Sample solution (500 µL) was removed at set times and replaced with the same volume of fresh HEPES buffer. Organic solvent (methanol:isopropanol = 1:3) was added to the sample (1:1 (*v/v*)) to break the liposomes and the DOX concentration was determined from its fluorescence using a microplate reader (ALVO series, PerkinElmer Inc.; ex/em, 480/595 nm). The release of DOX and DOX-loaded liposomes was determined as previously described with modification [43] and is based on quenching the DOX fluorescence. The values which were more than 100% were regarded as 100%.

## 4. Conclusions

In conclusion, we fabricated 3D-printed patches composed of F-GelMA, a semi-synthetic biocompatible polymer. Although in vivo experiments are necessary to demonstrate the therapeutic efficacy of these patches in treating cancer, patches prepared using a 3D bioprinter will be useful for implantation and may be applicable for treating cancer tissues in patients or in sites of surgical removal. We showed that the low viscosity of F-GelMA was greatly increased by the addition of CMC, and the increase in liposome particle size in F-GelMA hydrogels was inhibited by the addition of CMC. These results indicate that CMC is useful for adjusting the properties of printer ink and is a useful and safe pharmaceutical excipient in drug formulations. We also showed that drug release from 3D-printed patches was dependent on the patch shapes and UV exposure time, and that drug release can be controlled. Taken together, the present results provide useful information for the preparation of 3D printed objects containing liposomes and other nanoparticle-based nanomedicines.

## Figures and Tables

**Table 1:** Printing conditions of patches.

3D Geometries	Nozzle Diameter	Pressure
Cylinder	22 G	65
Torus	22 G	65
Gridlines	27 G	70

## Supplementary Materials

The following are available online at https://www.mdpi.com/1660-3397/18/6/325/s1, Figure S1: Stability of PEGylated liposomal DOX. (a) Typical size distribution of PEGylated liposomal doxorubicin collected from different hydrogel formulations. (b) The mean particles size and PDI of PEGylated liposomal doxorubicin collected from different hydrogels.
