# Supplementary material for: Fabrication of 3D-Printed Fish-Gelatin-Based Polymer Hydrogel Patches for Local Delivery of PEGylated Liposomal Doxorubicin
Source: Mar Drugs. 2020 Jun 20;18(6):325. doi: 10.3390/md18060325 (PMC7344981; doi:10.3390/md18060325)
Supplement: Supplementary file 1 [file marinedrugs-18-00325-s001.pdf]

marine drugs

Supplementary Materials:

Fabrication of 3D-Printed Fish-Gelatin-Based Polymer Hydrogel Patches for Local Delivery of PEGylated Liposomal Doxorubicin

Jin Liu, Tatsuaki Tagami and Tetsuya Ozeki *

Drug Delivery and Nano Pharmaceutics, Graduate School of Pharmaceutical Sciences, Nagoya City University, 3-1 Tanabe-dori, Mizuho-ku, Nagoya, Aichi 467-8603, Japan

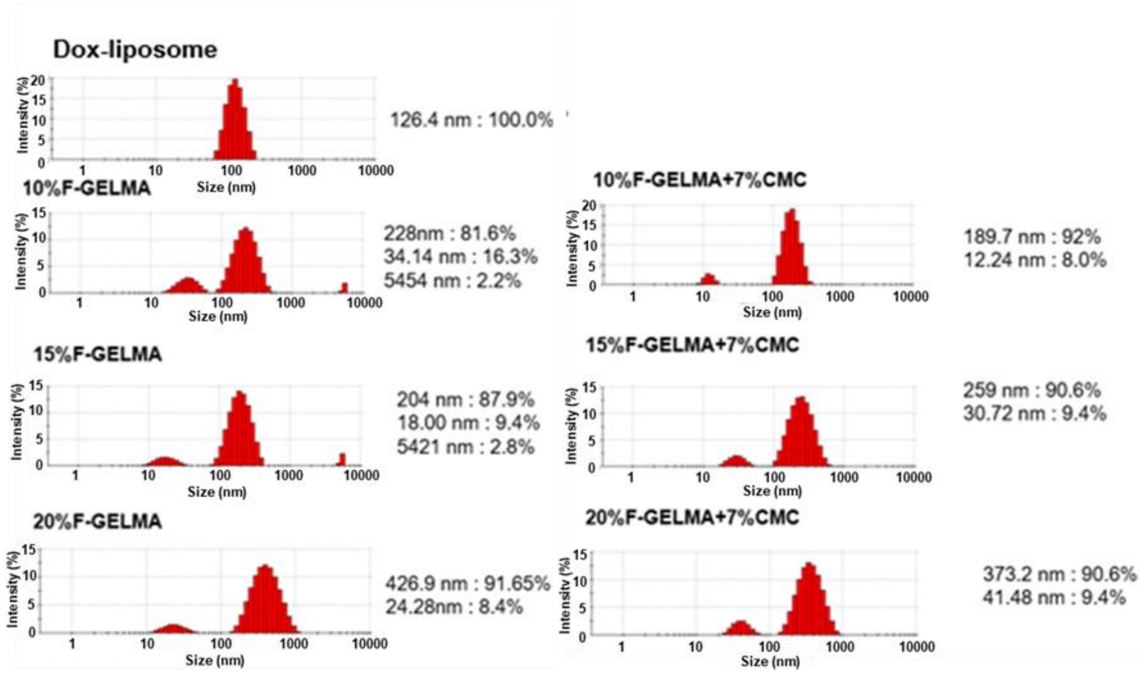

(**a**)

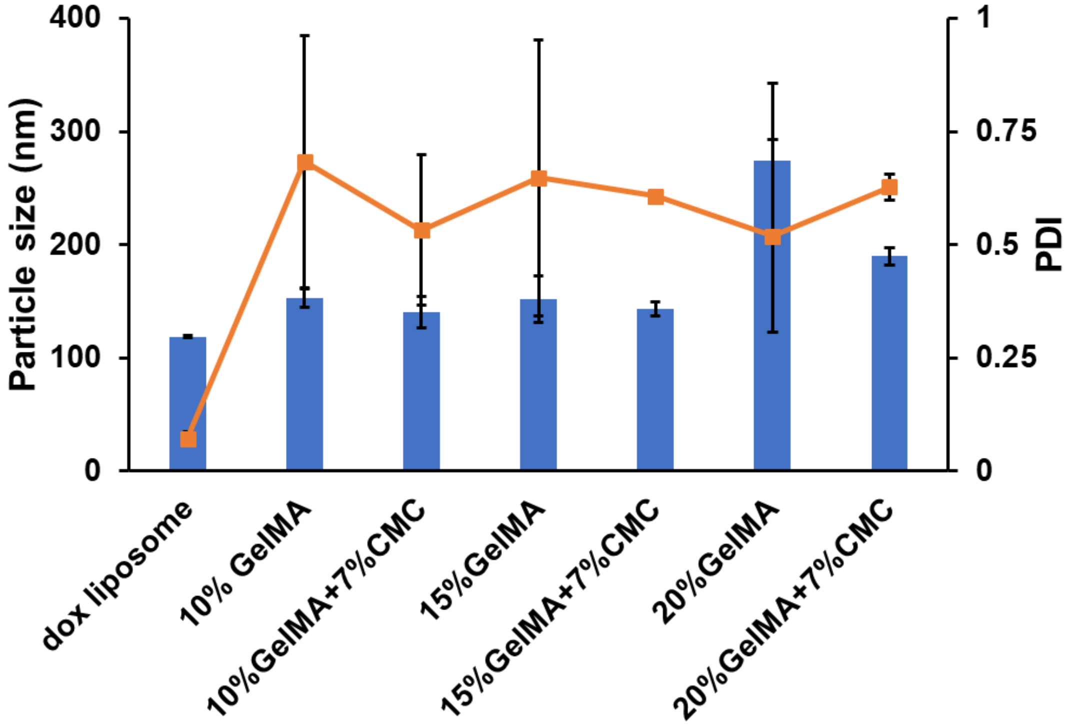

(**b**)

Figure S1. Stability of PEGylated liposomal DOX. (**a**) Typical size distribution of PEGylated liposomal doxorubicin collected from the different hydrogel formulations. (**b**) The mean particles size and PDI of PEGylated liposomal doxorubicin collected from different hydrogels. The data represent the mean ± SD.
